# Supplementary material for: Mitochondrial dynamics can resolve stoichiometric challenges in plant mtDNA repair
Source: bioRxiv. 2026 Oct 9:2026.10.08.757513. Preprint. [Version 1] doi: 10.64898/2026.10.08.757513 (PMC13692242; doi:10.64898/2026.10.08.757513)
Supplement: Supplementary file 1 [file NIHPP2026.10.08.757513v1-supplement-1.pdf]

bioRxiv preprint doi: https://doi.org/10.64898/2026.10.08.757513; this version posted October 9, 2026. The copyright holder for this preprint (which was not certified by peer review) is the author/funder, who has granted bioRxiv a license to display the preprint in perpetuity. It is made available under a CC-BY 4.0 International license.

Supplementary Information

Estimating rates of (mt)DNA damage and repair

Formation of apurinic/apyrimidinic sites, which often convert to single strand breaks, have been measured at around 1.5 sites per 10^6 bases per day under (human) physiological conditions (Nakamura et al., 1998).

(Saul & Ames, 1986) find: "Based upon measurements of DNA oxidation products in human urine, we estimate that the total rate of formation of all oxidative DNA damage products… is on the order of a few thousand events per cell per day." This (given around 3×10^9 bases of DNA in the human cell) gives roughly the same scale of estimate of 1 damaged product per 10^6 bases per day.

(Crine & Verly, 1976) observed that T7 phage DNA in a model of intracellular medium experienced 0.018 SSBs per hour. This was (given about 40 kb in T7 phage DNA) cited with a characteristic SSB timescale of 2.5 h per 10^6 dsDNA bases in (Shapiro, 1981) – in a table summarising evidence of other modes of damage, of which SSBs are the most common. This figure – roughly 10 damage events per 10^6 bases per day -- was taken forward as 55 200 SSBs per human nuclear DNA per cell per day in the widely cited (Tice & Setlow, 1985).

Which is the more appropriate class of figure for plant mtDNA? mtDNA is less packaged and protected than nuclear DNA, but likely more so than viral DNA. In many animals, mtDNA accumulates mutations several-fold more rapidly than nuclear DNA (although much of this might be due to differences in repair rates and not solely differences in damage rates (Broz et al., 2025)). We might therefore expect a figure towards the higher end of this range. For BER, we by default adopt a rate of 1 damage event per mtDNA per day – as the plant mtDNA genome is typically several hundred kb (though individual mtDNA molecules may be smaller), this corresponds roughly to the 10 events per 10^6 bases per day.

For template repair, we consider the rate at which mismatches form due to mtDNA replication. Eukaryotic DNA replication typically involves 1 mismatch every 10^4-10^5 bases (Kunkel & Erie, 2015), with in vitro measurements of plant mtDNA polymerase showing similar error rates (Ayala-García et al., 2018). Plant mtDNA turnover can be fast, on the scale of 1 day (Dai et al., 2005), though in other tissues this process is likely on a slower order of magnitude (Bendich, 2010). In that case, we might expect around 0.1 events per mtDNA genome per day, with this value being highly dependent on the amount of mtDNA replication (if any) occurring in the tissue.

According to (Ahmadi et al., 2018) and (Ahmadi et al., 2021), the scanning speed of DNA glycosylases and DNA endonuclease can be around 0.64 $\mu m^2/s$. With base pair spacing around 0.34 nm per bp, 1 μm corresponds to around 3000 base pairs, so the diffusion rate is around 5×10^6 bp^2/s. The characteristic timescale needed to cover L = 300 kb of DNA with this diffusion constant D is given by $L^2 / 2D = 9 \times 10^3$ s, or several hours. Of course, sliding, unbinding, and non-local motion may change these dynamics beyond

bioRxiv preprint doi: https://doi.org/10.64898/2026.10.08.757513; this version posted October 9, 2026. The copyright holder for this preprint (which was not certified by peer review) is the author/funder, who has granted bioRxiv a license to display the preprint in perpetuity. It is made available under a CC-BY 4.0 International license.

those expected from simple 1D diffusive search. We explore several values for the timescale of the initial damage recognition based on this general scale.

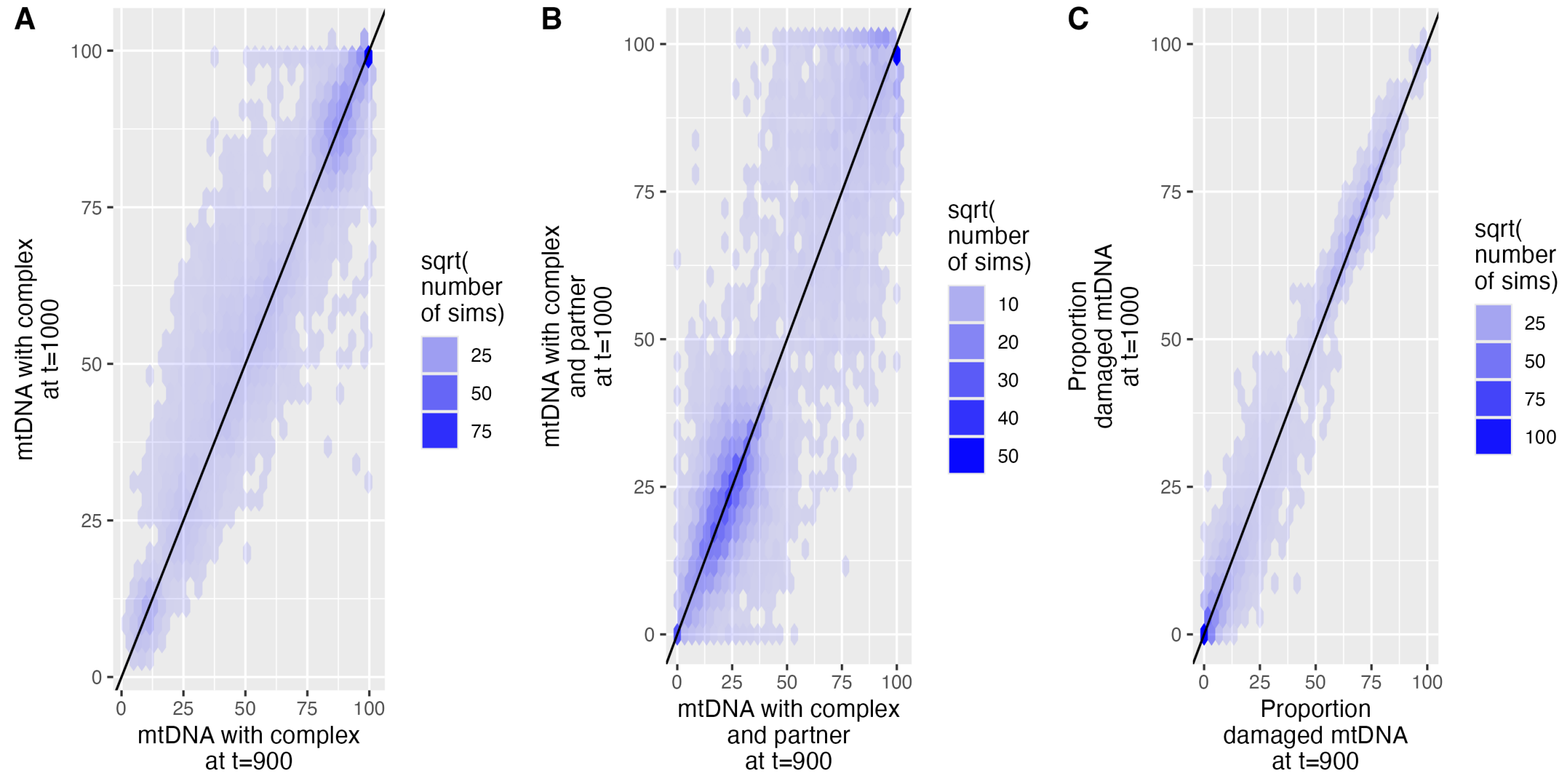

Supplementary Figure 1. **Stability of long-term simulation results. (A)** DNA-complex count, **(B)** templating capacity, **(C)** proportion of damaged mtDNA at t=900 h and t=1000 h after the start of the simulation. For each quantity, there is strong and symmetric agreement between the earlier and later observations, suggesting stabilisation of the system's behaviour.

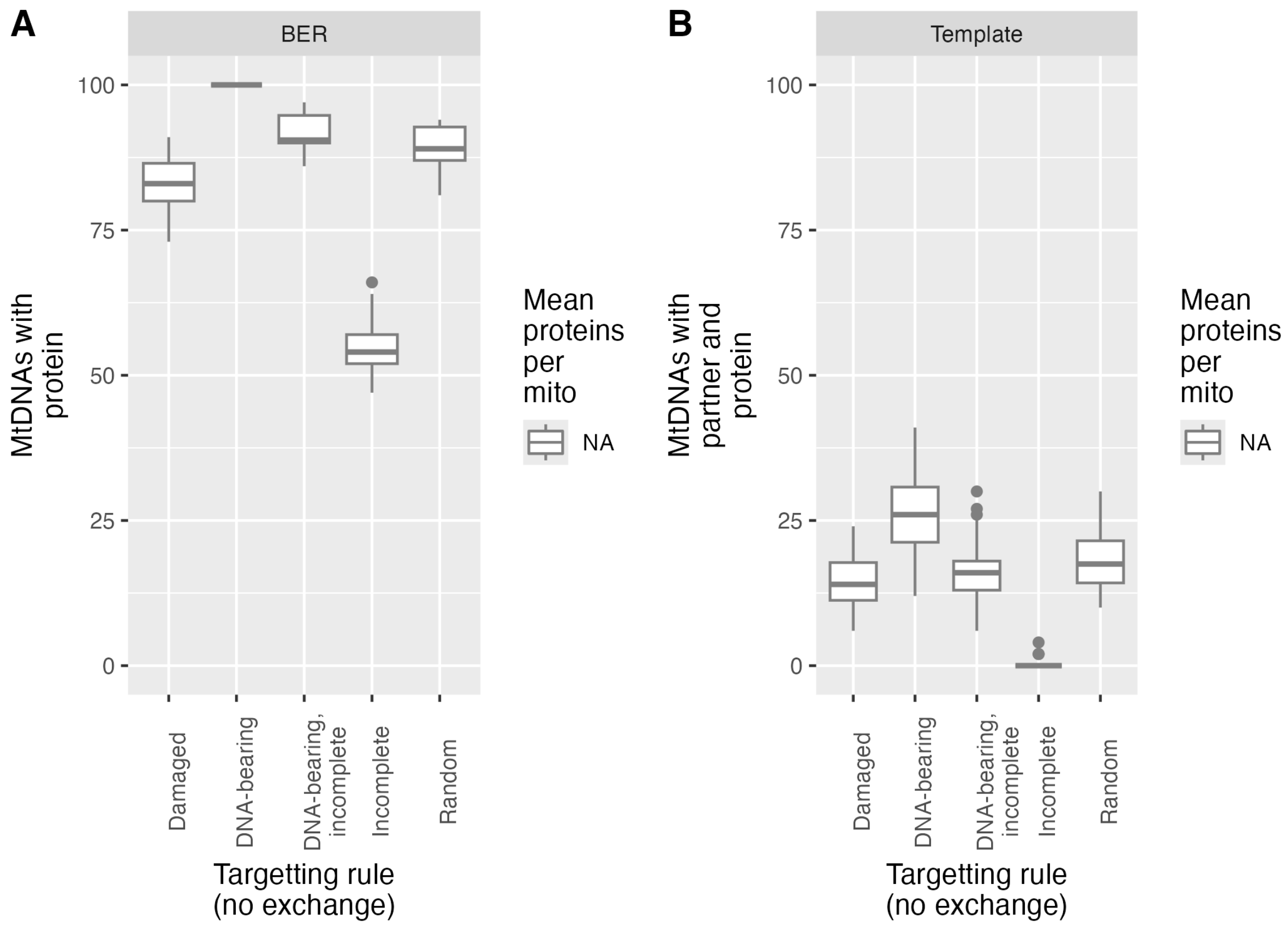

Supplementary Figure 2. **Impact of targetting rules on DNA-complex count and templating capacity. (A)** DNA-complex count (where a DNA-complex is an mtDNA and one copy of each of the five limiting proteins in the repair pathway) and **(B)** templating capacity (mtDNAs with a dimer of the repair protein and a partner mtDNA), under different targetting rules while fusion-based exchange is

bioRxiv preprint doi: https://doi.org/10.64898/2026.10.08.757513; this version posted October 9, 2026. The copyright holder for this preprint (which was not certified by peer review) is the author/funder, who has granted bioRxiv a license to display the preprint in perpetuity. It is made available under a CC-BY 4.0 International license.

absent. There are 100 mtDNAs in total (so the y-axis can be read as a percentage), and an average 0.33 per mitochondrion. Two parameterisations are used, corresponding to an average of 2 and 4 proteins of each type per mitochondrion.

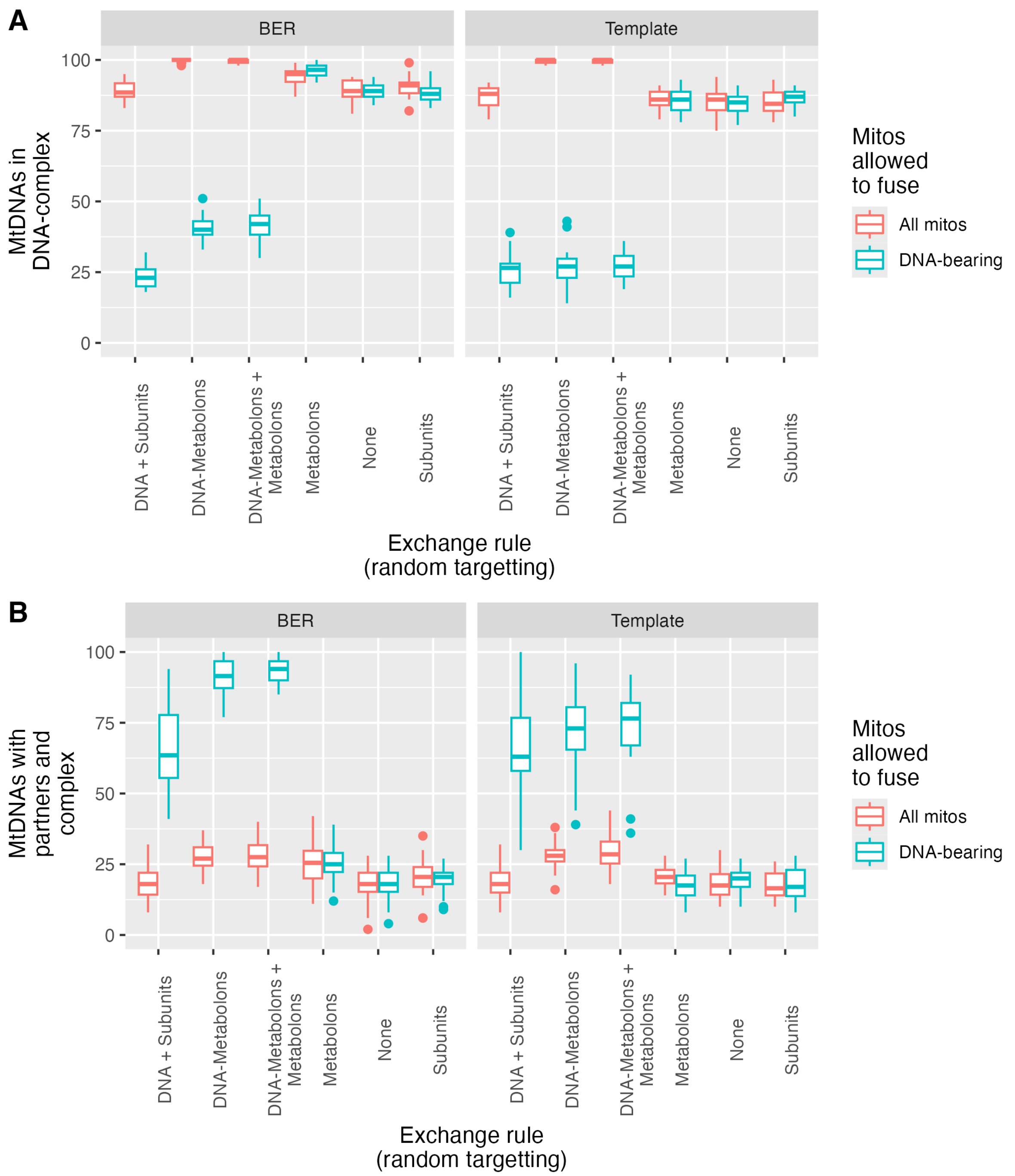

Supplementary Figure 3. **Impact of exchange rules on DNA-complex count and templating capacity. (A)** DNA-complex count (where a DNA-complex is an mtDNA and one copy of each of the five limiting proteins in the repair pathway) and **(B)** templating capacity (mtDNAs with a dimer of the repair protein and a partner mtDNA), under different exchange rules with random import targetting. There are 100 mtDNAs in total (so the y-axis can be read as a percentage), and an average 0.33 per mitochondrion. Two fusion rules are used: all mitochondria can fuse, or only mitochondria bearing mtDNA.

bioRxiv preprint doi: https://doi.org/10.64898/2026.10.08.757513; this version posted October 9, 2026. The copyright holder for this preprint (which was not certified by peer review) is the author/funder, who has granted bioRxiv a license to display the preprint in perpetuity. It is made available under a CC-BY 4.0 International license.

Stoichiometries and turnovers of repair proteins

(Ferrando et al., 2019) describes a base-excision repair pathway involving five steps. Of the proteins involved in these five steps that are found in (Fuchs et al., 2020), the minimal copy number element for each step is:

Step	Protein	Per-mito copy number
Glycosylase	APS3 Pseudouridine synthase/archaeosine transglycosylase-like family protein	4.79
Endonuclease/polynucleotide kinase (PNK)/phosphatase	Mitochondrial nuclease 1 (MNU1); Putative endonuclease or glycosyl hydrolase	4.25
dRP lyase	(none found)	
DNA polymerase	Organellar DNA polymerase gamma I (POLGAMMA1)	2.48
DNA ligase	DNA ligase 1 (LIG1)	42.0

MSH1 is a key player in plant mtDNA recombination and is likely to play a central role in templated repair (Broz et al., 2022, 2024; Wu et al., 2020; Zwonitzer et al., 2024). (Fuchs et al., 2020) find only two MSH1 copies per mitochondrion. The protein likely acts in dimeric form (Fuchs et al., 2020; Sloan et al., 2025) (Supp. Fig. 5).

(Nelson et al., 2013) looked across proteins and found, with substantial heterogeneity, an average detected protein turnover rate around ~ 0.1 per day.

bioRxiv preprint doi: https://doi.org/10.64898/2026.10.08.757513; this version posted October 9, 2026. The copyright holder for this preprint (which was not certified by peer review) is the author/funder, who has granted bioRxiv a license to display the preprint in perpetuity. It is made available under a CC-BY 4.0 International license.

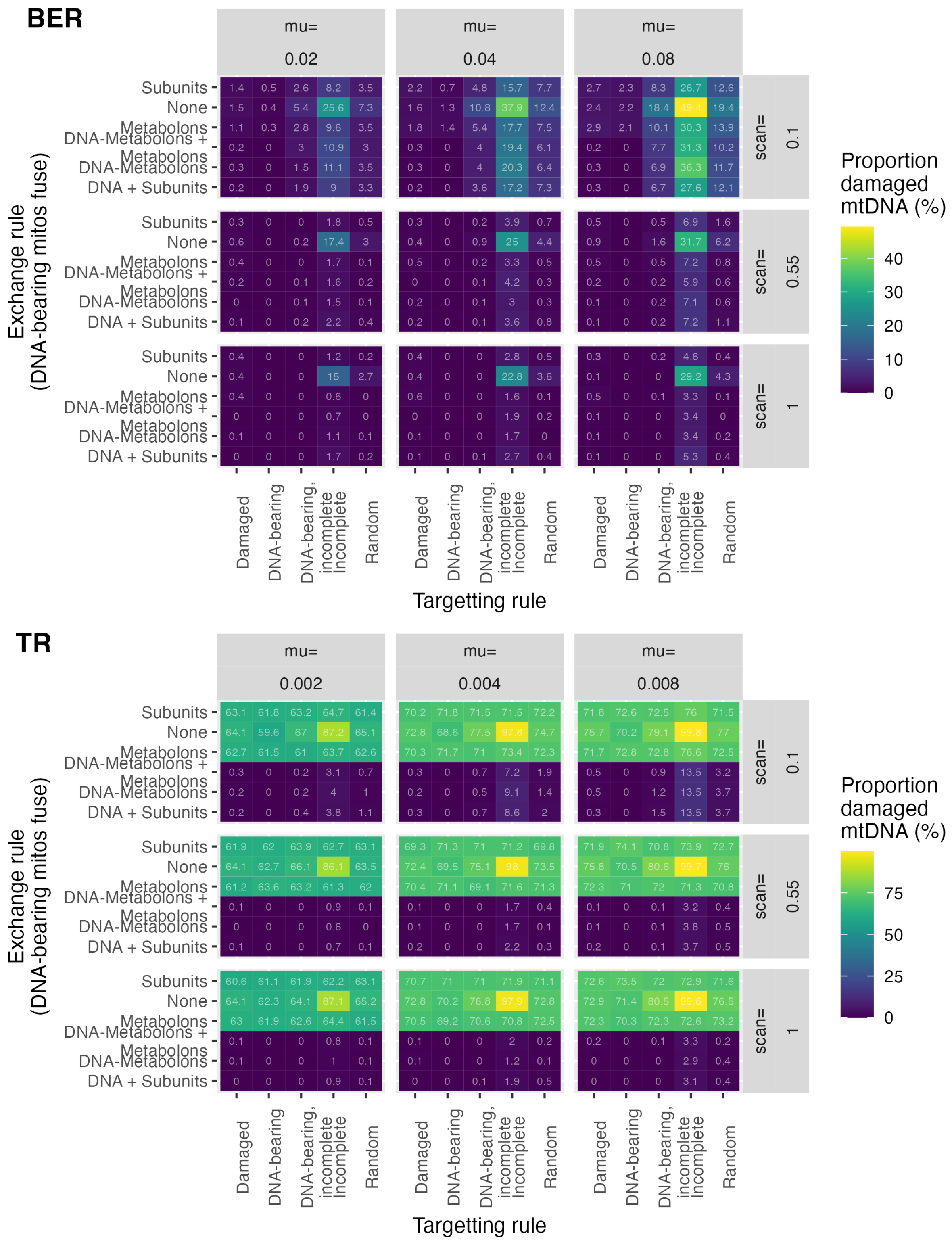

Supplementary Figure 4. Influence of damage rate (“mu”, per hour) and scanning rate (“scan”, per hour) for (top) BER pathway; (bottom) template repair pathway. Model damage rates are an order of magnitude lower in the TR experiments (see text). Each panel gives a combination of these parameters; each pixel in a panel (as in Fig. 5) gives the proportion of damaged mtDNA under those conditions.

bioRxiv preprint doi: https://doi.org/10.64898/2026.10.08.757513; this version posted October 9, 2026. The copyright holder for this preprint (which was not certified by peer review) is the author/funder, who has granted bioRxiv a license to display the preprint in perpetuity. It is made available under a CC-BY 4.0 International license.

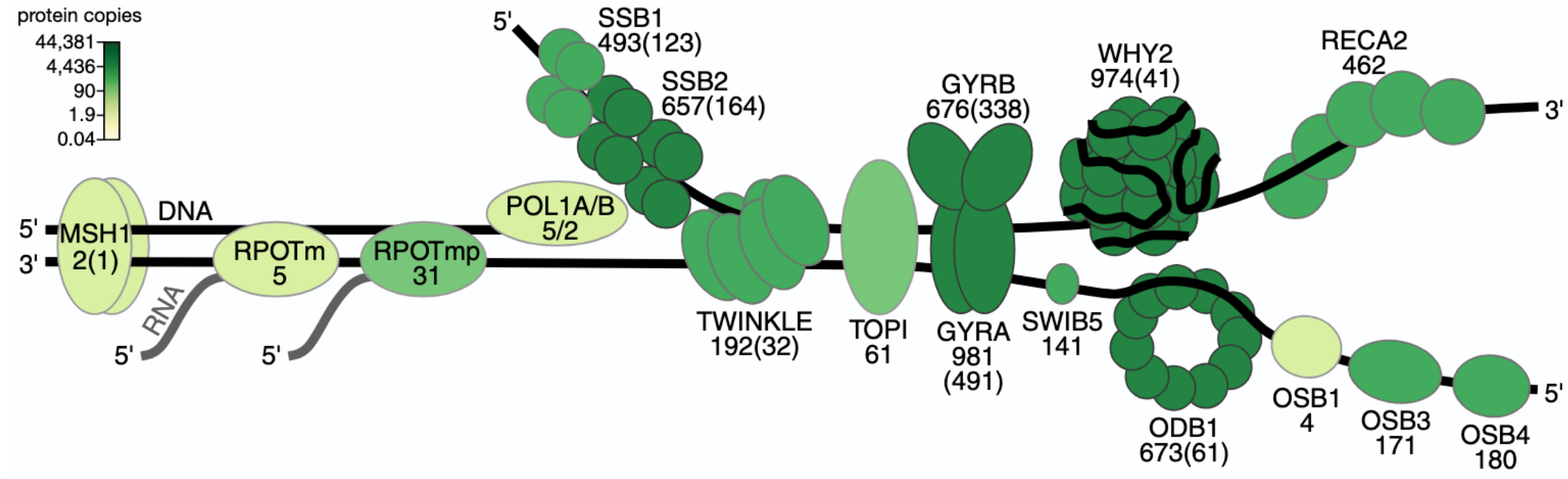

Figure 5. Proteins associated with the mitochondrial genome.
DNA-binding proteins detected are represented as binding to single-stranded or double-stranded DNA (based on findings recently reviewed by Gualberto and Kühn, 2014; Gualberto and Newton, 2017). The DNA polymerases 1A (POL1A) and 1B (POL1B) are summarized by a single symbol. For proteins known or assumed to be present in homo- or hetero-oligomers the inferred number of complexes is shown in brackets. For DNA gyrase composed of two DNA gyrase A (GYRA) and two DNA gyrase B (GYRB) subunits each, the subunits are of similar abundance (981 versus 676), which allows estimating about 400 enzyme complexes per mitochondrion. RecA homolog 2 (RECA2); MutS homolog 1 (MSH1); organellar DNA-binding protein 1 (ODB1); organellar single-stranded DNA-binding protein (OSB1, OSB3, OSB4); T3/T7 bacteriophage-type RNA polymerase, mitochondrial (RPOTm); T3/T7 bacteriophage-type RNA polymerase, mitochondrial/plastidial (RPOTmp); mitochondrial single-stranded DNA-binding protein (SSB1, SSB2); SWI/SNF protein complex B protein 5 (SWIB5); Type-IA DNA topoisomerase (TOPI), twinkle-like DNA primase-helicase (TWINKLE); Whirly 2 (WHY2). Not included in the figure: homolog of bacterial RecG (RECG1, four copies), DNA repair protein RadA-like protein (RADA, 34 copies); protein related to *Escherichia coli* RuvC, involved in homologous recombination (Gualberto and Newton, 2017) (eight copies); DNA Ligase I (47 copies).

Supplementary Figure 5. Per-mitochondrion copy numbers of mtDNA-associated protein from (Fuchs et al., 2020) (under CC-BY).
